# Stochastic and Self-Organisation Patterns in a 17-Year PM_10_ Time Series in Athens, Greece

**DOI:** 10.3390/e23030307

**Published:** 2021-03-05

**Authors:** Dimitrios Nikolopoulos, Aftab Alam, Ermioni Petraki, Michail Papoutsidakis, Panayiotis Yannakopoulos, Konstantinos P. Moustris

**Affiliations:** 1 Department of Industrial Design and Production Engineering, University of West Attica, GR-12244 Aigaleo, Greece; mipapou@uniwa.gr; 2 Centre for Earthquake Studies, National Centre for Physics, Islamabad 44000, Pakistan; aftab.alam@ncp.edu.pk; 3 Department of Informatics and Computer Engineering, University of West Attica, GR-12233 Aigaleo, Greece; epetraki@uniwa.gr (E.P.); pyian@uniwa.gr (P.Y.); 4 Department of Mechanical Engineering, University of West Attica, Petrou Ralli & Thivon 250, GR-12244 Aigaleo, Greece; kmoustris@uniwa.gr

**Keywords:** air pollution, PM10, Statistical analysis, Boltzmann entropy, Tsallis entropy

## Abstract

This paper utilises statistical and entropy methods for the investigation of a 17-year PM_10_ time series recorded from five stations in Athens, Greece, in order to delineate existing stochastic and self-organisation trends. Stochastic patterns are analysed via lumping and sliding, in windows of various lengths. Decreasing trends are found between Windows 1 and 3500–4000, for all stations. Self-organisation is studied through Boltzmann and Tsallis entropy via sliding and symbolic dynamics in selected parts. Several values are below −2 (Boltzmann entropy) and 1.18 (Tsallis entropy) over the Boltzmann constant. A published method is utilised to locate areas for which the PM_10_ system is out of stochastic behaviour and, simultaneously, exhibits critical self-organised tendencies. Sixty-six two-month windows are found for various dates. From these, nine are common to at least three different stations. Combining previous publications, two areas are non-stochastic and exhibit, simultaneously, fractal, long-memory and self-organisation patterns through a combination of 15 different fractal and SOC analysis techniques. In these areas, block-entropy (range 0.650–2.924) is significantly lower compared to the remaining areas of non-stochastic but self-organisation trends. It is the first time to utilise entropy analysis for PM_10_ series and, importantly, in combination with results from previously published fractal methods.

**Data Set License:** license under which the dataset is made available (CC0, CC-BY, CC-BY-SA, CC-BY-NC, etc.)

## 1. Introduction

Urban air pollution is a related problem for the sustainable environment, human society and economy [1,2]. Imposed by human intervention, urban air pollution is now a sustained part of the urban environment, governed by mechanisms such as emission, dilution and transportation [3]. Significant urban pollutants are the sulphur dioxides (SO_2_), nitrogen oxides (NO_x_), ozone (O_3_) and the respirable particulate matter [4,5,6,7]. The term particulate matter (PM) refers to a mixture of solid particles and liquid droplets hovering in the air close to the ground level (human respiratory height) or even higher. Airborne particulate matter represents a complex mixture of organic and inorganic substances. Some particles, such as dust, dirt, soot or smoke, are large or dark enough to be seen with the naked eye. Other particles are so small that they can only be detected using an electron microscope. Moreover, PM particles are not all of the same shape. Due to these differences and variations between PM particles, the term aerodynamic diameter is introduced. The aerodynamic diameter of a particle is defined as that of a sphere of density 1 $g/cm^{3}$ (density of water), which settles in still air at the same velocity as the particle in question [8]. Taking into account this definition, PM with an aerodynamic diameter less than or equal to 10 μm is referred as PM_10_, PM with an aerodynamic diameter less than or equal to 5.0 μm is referred as PM_5_, PM with an aerodynamic diameter less than or equal to 2.5 μm is referred as PM_2.5_, etc.

PM come in many different sizes and shapes and can be made up of hundreds of different chemicals. Some of them are emitted directly from a source, such as construction sites, unpaved roads, fields, smokestacks, fires, etc. Most PM is formed in the atmosphere as a result of complex chemical reactions of substances such as sulphur dioxides (SO_2_) and nitrogen oxides (NO_x_). These pollutants are emitted from power plants, industries and vehicles. It is obvious that the size of PM (especially the one of PM_10_) is crucial to cause adverse health effects in people living in large cities and urban areas in general. This is due to the easiness by which PM_10_ enter the human body through the respiratory system and the upper respiratory tract. During the last decades, many scientists all over the world have investigated the health impact due to PM exposure [9,10,11,12,13,14,15,16,17,18,19]. Furthermore, the scientific research of the topic focuses on understanding the variations of urban air pollutants with time, by investigating the contribution of the environmental and meteorological parameters to the corresponding variations and modelling and forecasting future alterations [20,21,22,23,24,25,26].

Alternative approaches in PM_10_ investigation involve concepts that have their origins in the science of complex dynamical systems. Diverging techniques based on chaos have been applied to investigate the inter-state transitions of PM and several other urban air pollution systems. In particular, Kai et al. [27] reported two power-laws in three air pollution time series in Shanghai, China, indicating different self-organised critical (SOC) states. Liu et al. [28] employed detrended fluctuation analysis (DFA) and multifractals to investigate the temporal variations of the concentrations of SO_2_, NO_2_ and PM_10_ in the city of Shanghai, China. They reported that the corresponding air pollution was a self-organised critical (SOC) process. They observed different SOC behaviour within the SO_2_, NO_2_ and PM_10_ concentration time series, that have been attributed to differences in the corresponding power-law relations. Varotsos et al. [29] observed power-law correlations and persistent behaviour of deseasonalised ozone concentration time series with lags between one week and five years. Lee et al. [30] reported scaling invariance and decreasing box dimension function in hourly average of O_3_ time series concentrations during a whole year by analysing their data-series with monofractals. Windsor and Toumi [31] reported, through the method of R/S analysis, high persistency of hourly ozone concentration time series extending up to 400 days. Similarly, Weng et al. [32] confirmed through (R/S) analysis the nonlinearity, fractality, persistency and long-lasting characteristics of deseasonalised ground level peak ozone concentration time series in southern Taiwan. Xue et al. [33] concluded that R/S analysis, DFA, power-law spectral analysis, linear unbiased estimates, correlation analyses and power spectrum analysis are sophisticated approaches to analyse and describe the distribution, periodicity and trends of air pollutant concentrations. Similarly, Dong et al. [34] employed DFA and multifractal DFA in PM_2.5_ and PM_10_ data series and reported the statistical self-affinity, long memory and multifractality of their pollutant time series. Finally, Yuval and Broday [35] employed continuous wavelet transform fractal analysis and found that the predictability of air pollutants is consistent with that of meteorological variables in the short scale.

Up to now, the reporting team has employed different chaotic techniques to investigate and delineate the critical inter-change phases of PM_10_ pollutants in the Greater Athens Area (GAA) in Greece [36,37]. Detrended fluctuation analysis (DFA), rescaled-range (R/S) analysis and fractal-dimension (FD) analysis via three different methods (Higuchi, Katz and Sevcik) have been employed in lengthy 17-year PM_10_ time series in GAA. Most importantly, a new novel method has been introduced and applied in PM_10_ time series that is based on the combination of two or more chaotic techniques [36,37]. In [36], a combination of four techniques is reported (R/S analysis versus FD analysis via the methods of Katz, Higuchi and Sevcik). In [37], a combination of two techniques is employed (DFA versus R/S analysis); however, most significantly, a combination of 13 different chaos analysis techniques is reported. The combination of many different chaos analysis techniques have been employed and checked in critical electromagnetic time series [38] and critical radon in groundwater series [39]. The importance of the above approach is that it employs sliding windows forwarded one sample, covering, in that manner, a fine analysis of the time series.

As follows from the available scientific data of the topic, no investigation has targeted the entropy of urban air-pollution time series. Thus, the present paper focuses on the use of entropy analysis methods for the investigation of lengthy PM_10_ time series in GAA. Two significant measures of entropy are employed, namely the Boltzmann entropy (BE) and the Tsallis entropy (TE). Both techniques are employed in sliding windows and via symbolic dynamics [40,41,42], in selected parts. It is significant to note here that, since entropy measures the level of organisation of a system, a low order of fractality is related to a low order of entropy and vice versa. Hence, in the consensus of the above-mentioned publications of the reporting team, the use of entropy is a significant alternative viewpoint of the internal non-linear dynamics of the PM_10_ time series generation system in GAA. Based on a very recent paper of the team [43], which studies this time series with pure statistical methods, the present paper employs statistical techniques to analyse the stochastic tendencies, as well. Similar to the previous studies, sliding windows are employed in the statistical analysis. This yields to analysis of monthly and annually periods of the PM_10_ system together with other stochastic tendencies. The underlying concept is that the PM_10_ of GAA is probably both stochastic and non-linear since it has pure stochastic-statistical parts and others with chaos behaviour and long memory. The study combines the stochastic and entropy techniques, in a similar approach as with the fractal and long-memory techniques. Where the PM_10_ system is out of stochastic behaviour, it is analysed for self-organised tendencies. Several periods are identified and discussed.

The analysis is performed on measurements recorded by the Department of Environmental Protection of the Hellenic Ministry of Environment and Energy at a sampling frequency of 1 $min^{-1}$. As with the previous two related papers [36,37], this paper employs the same sub-series of the whole dataset, namely the average daily concentrations during 2001–2018 from five different PM_10_ monitoring stations within the GAA [44]. This sub-series is considered more suitable for long-term analysis because it averages the intra-day variations and minimises, consequently, the overall bias to the estimations. It should also be mentioned that the analysis of PM_10_ air-pollution systems is a major concern of the scientific community with studies originating from many cities all over the world. Especially for Athens, the capital of Greece, many papers depict that PM_10_ are among the most harmful pollutants for its inhabitants [7,8,10,11,12].

In the next sections, the research methodology, results and outcomes of this paper are given and discussed. Section 2 presents the experimental methods, namely the area of study and the instrumentation of measurements. Section 3 provides the mathematical background that is further needed in data analysis. More analytically, Section 3.1 provides the statistical methods that are needed to study the stochastic behaviour of the series. Section 3.2 is a brief outline of the methods that are employed in the analysis of self-organisation and fractals. Section 3.3 provides general information on entropy while the specific methods that are employed are given in Section 3.4 and Section 3.5. The results and discussion are presented in Section 4. Due to the diversity and extent of the results, a step-by-step approach is utilised. In this view, the main contributions of this paper are the following: (A) The statistical trends of all the series are delineated. Towards this, the time evolution of the first four statistical moments is calculated through one- and two-month sliding windows and lumping of annual windows. Together, the box-plot-outlier statistical behaviour of the different stations are also reported. Through this procedure and for all station data:(A1) decreasing trends are reported during the last two decades; (A2) noteworthy discrepancies from the Gaussian behaviour are addressed for one- and two-month data analyses; (A3) the annually organised data follow the Gauss distribution; and (A4) deviations of the general statistical patterns are found in association with several outliers. Potential sources are outlined. Importantly. the LYK station is, unexpectedly, found to have similar patterns as ARI and MAR stations. (B) The time evolution of the self-organisation patterns of all stations via Boltzmann and Tsallis entropy is determined through sliding windows. The time-evolution plots are reported, and, from these, the segments with entropy values below critical thresholds are found. The dates of 66 two-month windows are tabulated. (C) The statistical patterns above critical values are found for all stations. Towards this, a previously published statistical methodology, based on the residual concentrations, is used. The corresponding plots are given and the corresponding ASCII data are extracted. (D) The statistical trends of all series are combined with the corresponding self-organisation patterns. Towards this, a previously published two-step meta-analysis methodology is applied to the ASCII data of Points (B) and (C). The dates of nine two-month windows are tabulated with: (D1) statistical patterns above critical values; and (D2) entropy measures below critical values, importantly, from the data of at least three stations simultaneously. (E) The results of Point (D) are compared with the results of the previous publications on the same times series. Of extreme importance is that the dates of three two-month windows are located that are common with the previous publications. The dates of these two-month windows are significant because 15 different techniques provide evidence of critical self-organisation and fractal trends. (F) For the two most significant areas of Point (E), block entropy analyses are reported versus the analyses of the remaining identified parts with SOC patterns. Significantly lower block entropy values are reported for the two areas identified with the combination of 15 techniques, compared to the remaining areas.

## 2. Experimental Methods

The urban complex of Athens and Piraeus (the port of Athens city) is a large urban area in the Attica Peninsula which includes Athens city, Piraeus city and their suburbs. It extends mainly to the Attica basin and within the administrative boundaries of the five regional units of the Attica Region: Central Sector of Athens, Southern Sector of Athens, Northern Sector of Athens, Western Sector of Athens and Piraeus. In a similar meaning, the term greater Athens area (GAA) is often used. According to the Greek National Census 2011, the GAA has a population of about 2.6 million inhabitants, which corresponds to 69% of the total population of the Attica Region [45]. The GAA basin is surrounded by high mountains. More specifically, on the west side of GAA, the mountain Egaleo (468 *m* a.s.l.) is located. To the north-northwest of GAA, there is Parnitha Mountain (1,413 *m* a.s.l.); to the north-north-east, there is Penteli Mountain (1,109 *m* a.s.l.); and on the east side, there is Ymittos Mountain (1,026 *m* a.s.l.). The only opening of GAA is located in the south region, the seaside of Saronic Gulf, which is a part of the Aegean Sea. The climate of the GAA is temperate and characterised as a typical Mediterranean climate. In general, sunny days are very common, even in winter. Rains occur mainly from October to April but over the whole year the rainfall amount is very low and does not exceed 400-450 mm. The mean minimum monthly temperature is about 7.0 °C and the mean maximum monthly temperature is about 31.8 °C [46]. Finally, the prevailing winds are south direction winds resulting in the coexistence of the surrounding mountains a major air pollution problem due to the bad air circulation and air pollutants dispersion [46].

**Figure 1:**
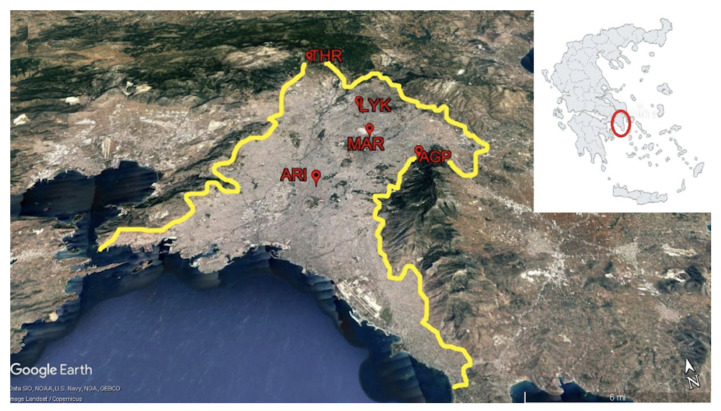
The map of the GAA and the location of the five air pollution monitoring stations.

The PM_10_ mean daily concentrations used in this work came from five air pollution monitoring stations (Figure 1 ) located in the GAA. These stations belong to the air pollution monitoring network which operates under the auspices of the Hellenic Ministry of Environment and Energy (HMEE). Pollutants are measured on a continuous basis 24 h a day. The response time of the automatic analysers is of the order of 1 min, i.e. each analyser gives a value approximately every minute. With a microprocessor, located in each automatic station and connected to the automatic analysers, the mean hourly values are calculated every hour. These hourly concentration values are transferred to the server of the service, through a telephone line, and in this way it is possible to continuously monitor the levels of air pollution in the area. Furthermore, the measuring technology of PM_10_ is based on the method known as absorption of β-radiation. The calibration of PM_10_ analysers is based on the absorption of β-radiation by a standard sample of known concentration [47]. Figure 1 depicts the map of the GAA and the location of the five air pollution monitoring stations. Table 1 shows the characteristics of the five examined air pollution monitoring stations.

## 3. Mathematical Methods

### 3.1. Statistical Methods

At first, statistics are used to explore the general trends of the mean daily PM_10_ concentrations recorded by the five examined air pollution monitoring stations within the GAA. To investigate the statistical trends of the data, sliding windows of different lengths are employed. In particular, windows of length 32 (approximately one-month data) are moved forwards with a step of 32 samples (one-month analysis lumped one month forwards) and with a step of 1 (one-month analysis slid-glided one day forwards). The latter analysis provides very fine tuning of the PM_10_ concentrations series, since every 32-length window contains 31 common samples with the previous and the following windows and only one different sample due to the sliding process [37]. The 32-length window with a one-step movement has been applied with success in a recent publication of the team for PM_10_ air pollution system [37]. As a step further, windows of 64 samples (approximately two-month duration) are utilised as well and moved one sample forwards, in the consensus of another recent publication of the team, also for PM_10_ time series [37]. In each window, the statistical parameters checked are the average value, standard deviation, kurtosis and skewness of the series, namely the first four statistical moments of the data under investigation. Moreover, windows of 365 samples (annual data) are also employed that are moved forwards 365 samples (lumping process). For this analysis, additionally, the *p*-value corresponding to the Shapiro–Wilk test is also derived to explore if the corresponding annual data follow the Gaussian distribution. The statistical tests are derived with R package.

### 3.2. Chaos and Self-Organisation Methods

Several physical systems exhibit complex non-linear behaviour described by chaos. While chaos leads to states that are very sensitive to the initial conditions of the system, there can be critical states (phase space solutions) that are combined in a manner that the system will pass through a series of such states via a complex solution curve that will emerge inevitably. Such states are found in fractal and self-organised systems.

Fractal systems reflect their chaotic behaviour when dilated, translated or rotated in space. Depending on their mathematical properties, these systems are characterised as self-affine or self-similar. Such systems are fractals because the self-affinity and the self-similarity characterise all parts of the system, in the manner that every part is a large- or small-scale imitation or representation of the whole system. Due to this property, the fractal systems can be investigated by analysing their parts. It is notable that scaling and fractal behaviour are associated with the system’s long memory [48,49] and complexity [48], where the complexity of a system indicates the existence of linear mechanisms and orderliness [48]. It is this complex orderliness that emerges as self-organisation of a system. When these self-organised states are connected with the corresponding chaotic critical fractal states, they represent the so-called self-organised critical (SOC) states. SOC and critical fractal states are closely related and also associated with the long memory of the system. For this reason, SOC analysis can reveal fractality and long memory and vice versa [50]. Systems in SOC states can show strong long-lasting associations connecting the past, presence and future of the system. In such critical states, the system will collapse through a series of states where its current state (presence) is affected not only by a previous state (deterministic approach) but also by a series of quite past states (long-lasting interactions of the past), and it also affects (inevitably) its future states in a long-lasting manner.

### 3.3. Entropy Analysis

Complex nonlinear dynamical systems can be viewed as information capacitors containing discrete series of messages [40,41]. Messaging is achieved by partitioning a continuous time series into a finite number of cells [42]. This procedure is often referred as “symbolic dynamics” [40,42]. Under symbolic dynamics, the actual continuous complex dynamics are viewed under finite precision and can be studied with the concept of entropy [40,50]. The related analysis can be implemented in sequential windows of a finite number of cells [50] (entropy analysis versus time) or in separated series blocks of symbols (block entropy analysis). There are several metrics of entropy that can be effectively utilised, to analyse the complexity of a system [51]. This is because large entropy values imply the existence of many different kinds of patterns and, therefore, high complexity and low organisation. On the other hand, decreased values of entropy (local minima), indicate increased organisation of patterns (local maxima) and, therefore, low order of complexity of a system.

In this paper, two types of entropy measures are employed, namely the Boltzmann entropy and the Tsallis entropy. These two measures are utilised as entropy analysis versus time and as block entropy analysis. They are described in detail below.

### 3.4. Entropy Analysis Versus Time

To implement entropy analysis versus time, the following steps are followed:

1. The time series is divided into windows of equal size, *n*.
2. The data of each window are sub-grouped in *N* equal-spaced bins.
3. In each window, the number of series samples, $m_{j},j\in \left[1,N\right]$, is counted that have values between zones, j−1 and *j*. The process is iterated for all possible *j*, i.e., it is implemented for every zone. If N>n, there will be zones that will not contain series values, namely $m_{i}=0$.
4. The probability, $P_{j}$, in zone *j* is calculated as $P_{j}=\frac{m_{j}}{n}$. If N>, there will be zones with zero probability, since $m_{j}$ is zero in such cases.
5. In every time series window i,i∈[1,n], the Boltzmann entropy is calculated as
  (1) $$H\left(i\right)=-k\sum_{j=1..N}P_{j}\cdot ln(P_{j})$$
  where *k* is the Boltzmann constant, i.e., $k=1.38064852\cdot 10^{-23}m^{2}kgs^{-2}K^{-1}$. Boltzmann entropy H(i) is a measure of uncertainty and gives the average amount of information needed to predict the actual distribution of measurements in window *i*. Tsallis entropy is calculated as
  (2) $$S_{q}\left(i\right)=k\frac{1}{q-1}\left(1-\sum_{j=1..N}p_{j}^{q}\right)$$
  where *k* is the Boltzmann constant and *q* is a real number measuring the non-extensivity of the system [51]. *k* is taken as 1.8 according to previous publications [40,41].

The procedure described in this section is a sliding-window technique. Thus, the present version of entropy analysis (versus time) refers to sliding windows. The reader should note that other researchers refer to this method as the gliding window technique [42,52].

### 3.5. Block Entropy Analysis

In block entropy analysis, the symbolic sub-sequences created by the symbolic organisation of a part of a time series are called words. The words are generated by selecting λ different letters from an alphabet and adopting certain block (word)-size, *n*, i.e., numbers of sequential letters that will be treated as a whole. Depending on λ and *n*, the maximum number, *N*, of different words in the selected alphabet can be determined. For example, for a λ=2 lettering, a threshold *C* is adopted. Each value above this threshold is then symbolised by a letter, e.g., 1, while each below by another, e.g., 0 [40,52]. Under this procedure, the initial time series of, for instance, length L=20, is transformed into 11001010111000101010 in this λ=2 lettering. The λ=2-letter symbolic time series can be read in discrete sets (lumping technique), of n=2 blocks, as (11|00|10|10|11|10|00|10|10|10|), where each block is one of the $N=2^{2}=4$ different words, i.e., (00, 01, 10,11), in this lettering [40]. Additionally, the above λ=2 letter sequence may be organised in blocks of n=3 letters with maximum of $N=N_{3,2}=n^{\lambda }=3^{2}=9$ different words, i.e., (000, 001, 010, 100, 110, 011, 010, 001, 111). Other sequences of words may be generated as well.

In general [40,52], a *L*-length time series is transformed, through symbolic dynamics, into a symbolic time series sequence, $\left[A_{1},A_{2}...A_{n}...A_{L}\right]$, composed by λ different letters, $\left[A^{1},A^{2}...A^{\lambda }\right]$, from a λ-length alphabet. Symbolic time series sequences are re-organised in *n*-sized word blocks composed by letters of the alphabet $\left[A^{1},A^{2}...A^{\lambda }\right]$. In linguistics, the word size is unconfined and, hence, linguistic words contain some or, potentially, all letters. On the other hand, in symbolic dynamics, the words are of fixed length *n*, n≥λ, and are chosen from $N_{\max}=N_{n,\lambda }=n^{\lambda }$ different fixed-sized words in the $\left[A^{1},A^{2}...A^{\lambda }\right]$ alphabet. In this way, the symbolic time series are re-organised as

$$...\underset{B_{1}}{\underbrace{A_{1}...A_{n}}}\underset{B_{2}}{\underbrace{A_{n+1}...A_{2n}}}...\underset{B_{i+1}}{\underbrace{A_{in+1}...A_{(i+1)n}}}...$$

blocks, where *i* is the consecutive number of the block, i.e., i=1...Totalnumberofblocks. In lumping, the *n* word blocks are sequentially independent sequentially dependent in sliding process. The probability of occurrence of a block, $\left[A_{1},A_{2}...A_{n}\right]$, of size *n* is calculated as

(3) $$p^{\left(n\right)}\left(A_{1},A_{2}...A_{n}\right)=\frac{\mathrm{Number}\;\mathrm{of}\;\mathrm{occurences}\;\mathrm{of}\;\mathrm{block}\;[A_{1},A_{2}...A_{n}]}{\mathrm{Total}\;\mathrm{number}\;\mathrm{of}\;\mathrm{blocks}}$$

In view of block entropy, Boltzmann block entropy is calculated as [40,52]

(4) $$H\left(n\right)=-k\sum_{\left(A_{1},A_{2},...A_{n}\right)}p^{\left(n\right)}\left(A_{1},A_{2},...A_{n}\right){\mathrm{lnp}}^{\left(n\right)}\left(A_{1},A_{2},...A_{n}\right)$$

where $p^{\left(n\right)}\left(A_{1},A_{2}...A_{n}\right)$ is the probability of occurrence of a sub-sequence of length *n* and *k* is the Boltzmann constant. The entropy H(n) is also a measure of uncertainty and gives the average amount of information necessary to predict a certain sub-sequence of length *n* [40].

Tsallis block entropy is calculated as [40,52]

(5) $$S_{q}\left(n\right)=k\frac{1}{q-1}\left(1-\sum_{\left(A_{1},A_{2},...A_{n}\right)}{\left[p^{\left(n\right)}\left(A_{1},A_{2}...A_{n}\right)\right]}^{q}\right)$$

where $p^{\left(n\right)}\left(A_{1},A_{2}...A_{n}\right)$ is the probability of occurrence of block $\left[A_{1},A_{2}...A_{n}\right]$ and, as above, *k* is the Boltzmann constant and q=1.8. As already mentioned, a high level of organisation is indicated when low values of Tsallis entropy are produced.

All types of block entropy analysis in this paper are calculated with λ = 2, *n* = 2–7, namely with two-letter symbolisation and 2–7 fixed-sized words.

## 4. Results and Discussion

**Figure 2:**
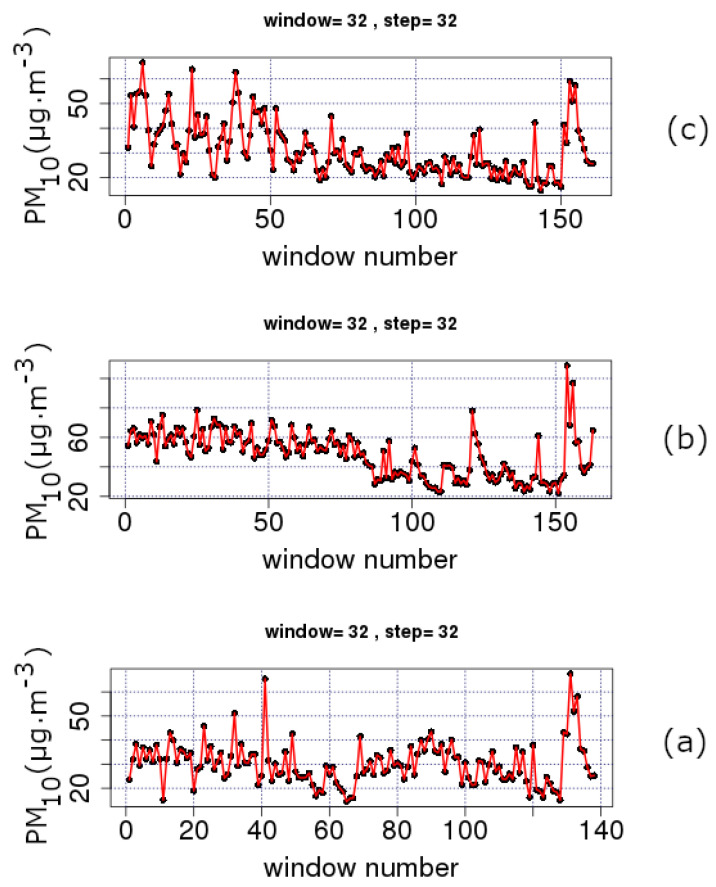
Average PM_10_ concentrations (μg/m^3^) for windows of length *n* = 32 and a step of 32 (one-month data lumped one month forward). GAA, period 2001–2010: (**a**) AGP series (**b**) LYK; and (**c**) THR series. PM_10_ concentration measurements in μg/m^3^. The diagrams are produced with R package.

**Figure 3:**
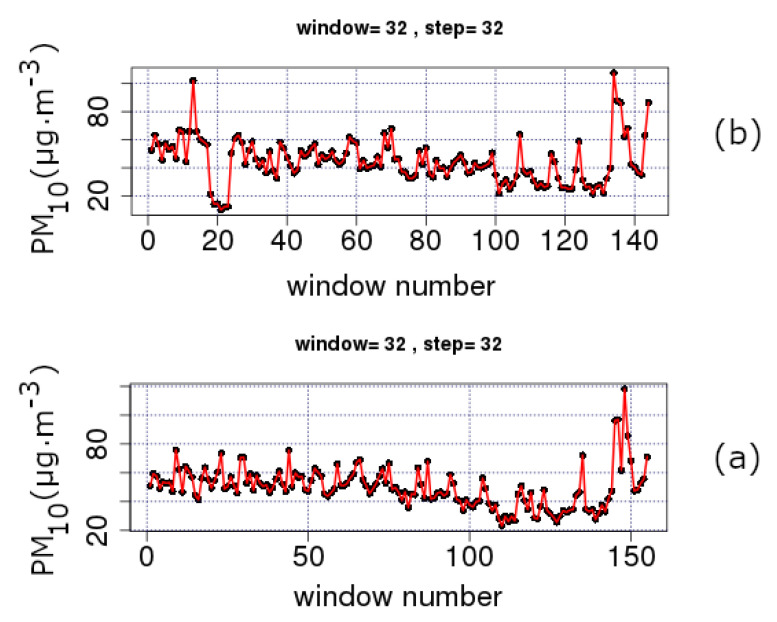
Average PM_10_ concentrations ($\mu g/m^{3}$) for windows of length *n* = 32 and a step of 32 (one month data lumped one month forward). GAA, period 2001–2010: (**a**) ARI series; and (**b**) MAR series. PM_10_ concentration measurements in $\mu g/m^{3}$. The diagrams are produced with R package.

Figure 2 and Figure 3 present the trends of PM_10_ concentration series according to the sliding window technique (please see Section 3.1). Each sub-figure corresponds to a station of Table 1 characterised by the HMEE as urban-traffic (UT) or suburban-background (SB), accordingly. It is important to mention that UT stations are characterised by a continuously built-up urban area with medium and high buildings in the manner that their pollution levels are mainly determined by the emissions from the nearby sources of traffic (roads, motorways and highways). On the other hand, SB stations are affected indirectly from all kind of sources of pollutants. Thus, they are not influenced by a certain source of pollution, but rather, collectively, by all nearby sources of pollution (industry, traffic, combustion, etc.). Referring to the data in Figure 2 and Figure 3, it should be noted that each data point corresponds to a segmented part of the series with *n* = 32 samples. Since the measurements are conducted on a daily basis, the *n* = 32 segment is the nearest power of two window to a monthly analysis. In this sense, Figure 2 and Figure 3 show the time evolution of, approximately, monthly data. The step in these figures is 32; therefore, there is a full coverage of the whole time series via lumping.

**Figure 4:**
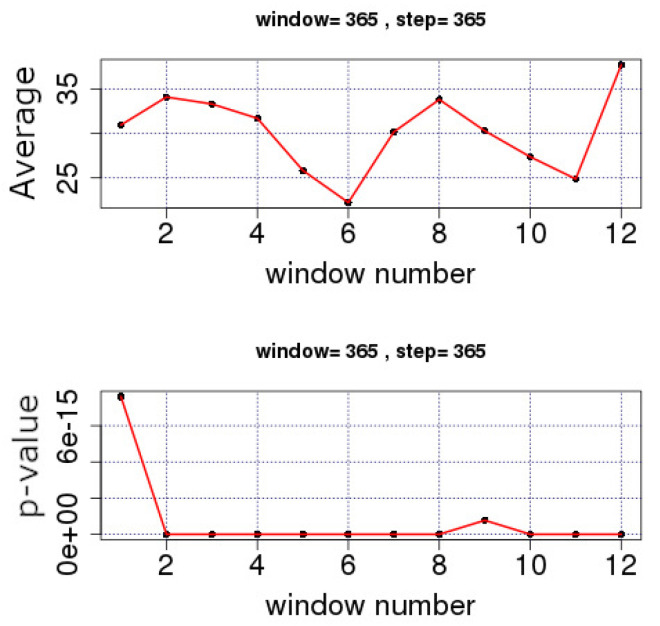
Average PM_10_ concentrations ($\mu g/m^{3}$) and p-value of the Shapiro–Wilk test for windows of length *n* = 365 and a step of 365 (one year data separated over the years). GAA, period 2001–2010, THR series. The diagrams are produced with R package.

**Figure 5:**
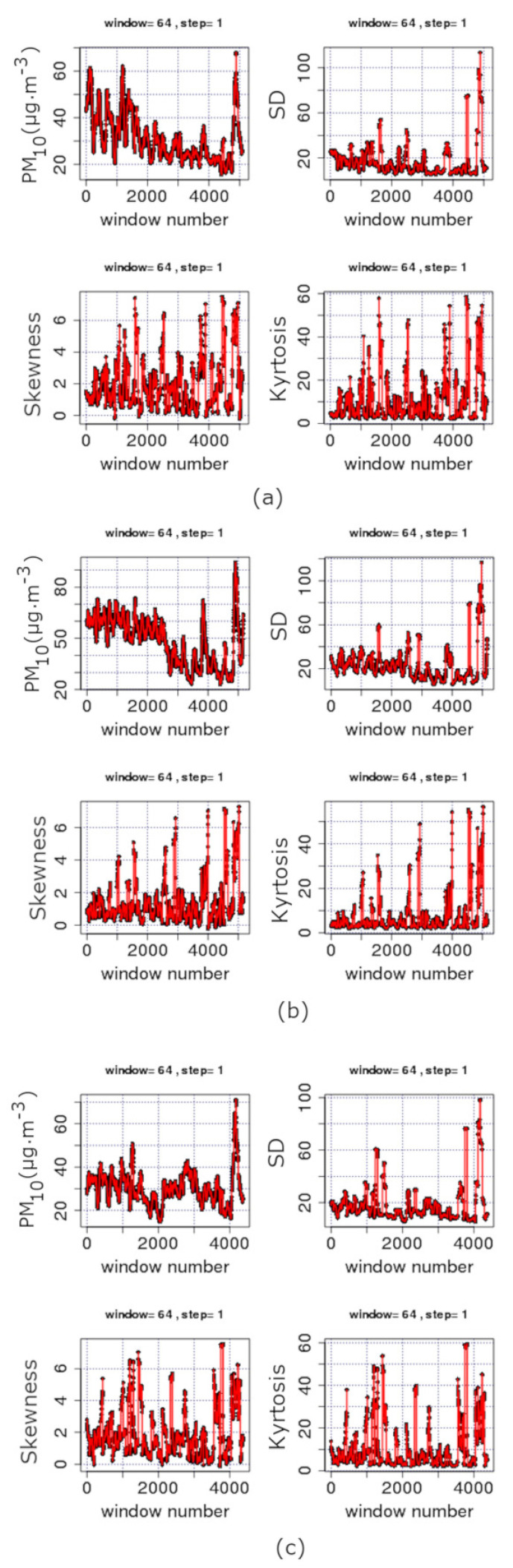
Time evolution of the first four statistical moments of segmented data of the time series for windows of length *n* = 64 with a step of 1. GAA, period 2001–2010: (**a**) AGP series; (**b**) LYK; and (**c**) THR series. Measurements in $\mu g/m^{3}$. The diagrams are produced with R package.

**Figure 6:**
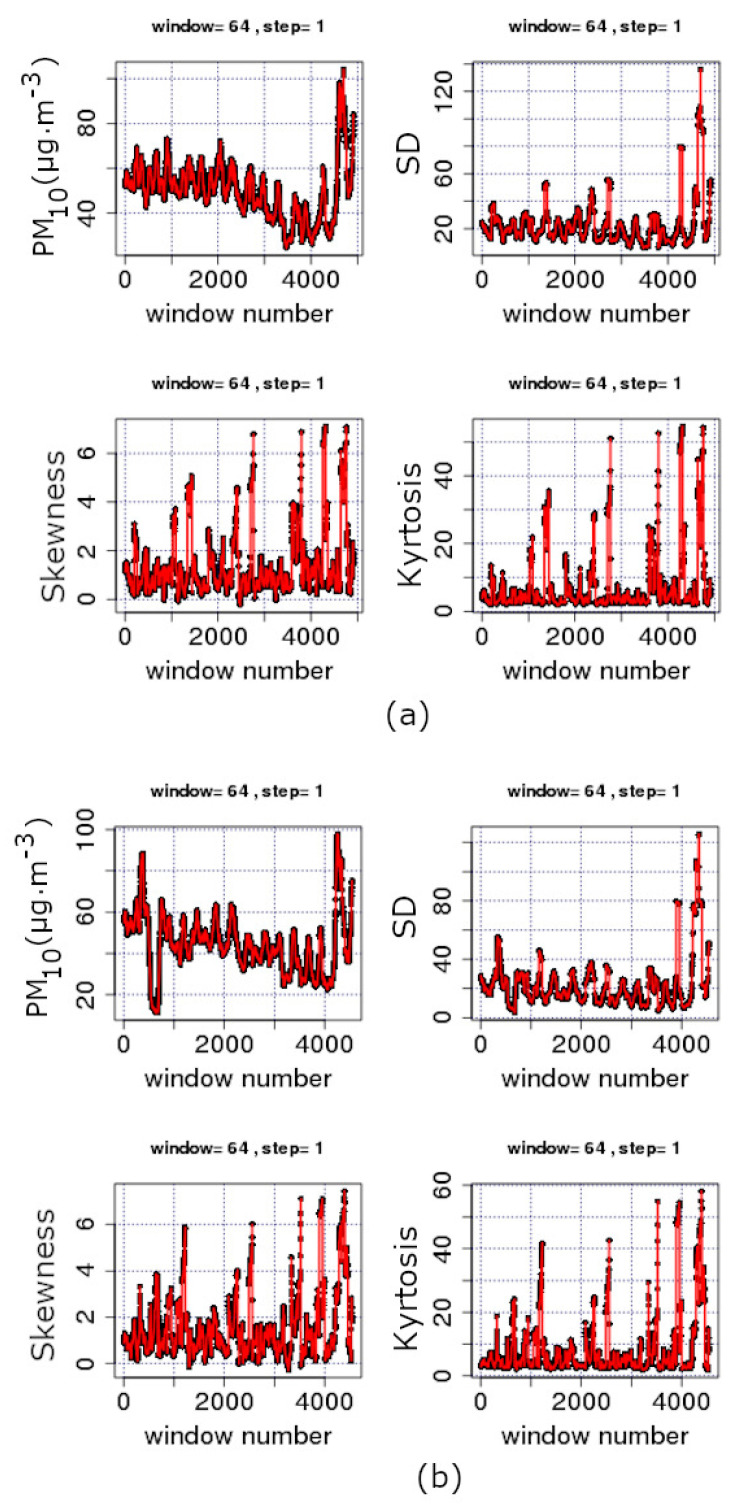
Time evolution of the first four statistical moments of segmented data of the time series for windows of length *n* = 64 with a step of 1. GAA, period 2001–2010: (**a**) ARI series; and (**b**) MAR series. Measurements in $\mu g/m^{3}$. The diagrams are produced with R package

All sub-figures of Figure 2 and Figure 3 show a clear decreasing trend during the last two decades (approximately Windows 40–130 depending on the completeness of data). This is more clearly observed in the example case of the THR station shown in Figure 4. The analysis of this figure is in segments of *n* = 365 samples with a step of 365, namely, the analysis is annual via lumping. The first order approximation of this trend is $PM_{c}=-0.76\cdot y+1573$ ($PM_{c}$ is the PM_10_ concentration and *y* is the year) and quantifies this decreasing trend. The low value of the square of the Spearman’s correlation coefficient of this first order approximation ($r^{2}$ = 0.5) is indicative of the multifaceted process of the PM_10_ generating system. This is also observed in Figure 5 and Figure 6 where the time evolution of the first four statistical moments of all investigated series is presented. When the data are analysed annually through lumping (Figure 4), the corresponding data follow the normal distribution since the p-value of the Shapiro–Wilk test is very low in all cases (even for the first peak which emerges mainly due to the start of the windowing process). On the other hand, when the data are analysed in segments of *n* = 64 (approximately two-month segmented data) glided with a step of 1, the observations provide a different viewpoint. Indeed, in Figure 5 and Figure 6, several skewness values are above 1, indicating that the corresponding parts of approximately two-month data (*n* = 64) do not follow the normal distribution. This is reinforced by the fact that there are several kurtosis values well above the normality kurtosis threshold value of 3. These findings imply, as mentioned above, that the process is multifaceted, and, hence, other mechanisms are present. These mechanisms, on the one hand, set the PM_10_ series away from normality. On the other hand, as indicated in two recent publications of the team [36,37], chaotic and SOC characteristics may also determine specific parts of the time series. In these time series parts the system is well out of the stochastic behaviour and, as a result, away of normality.

**Figure 7:**
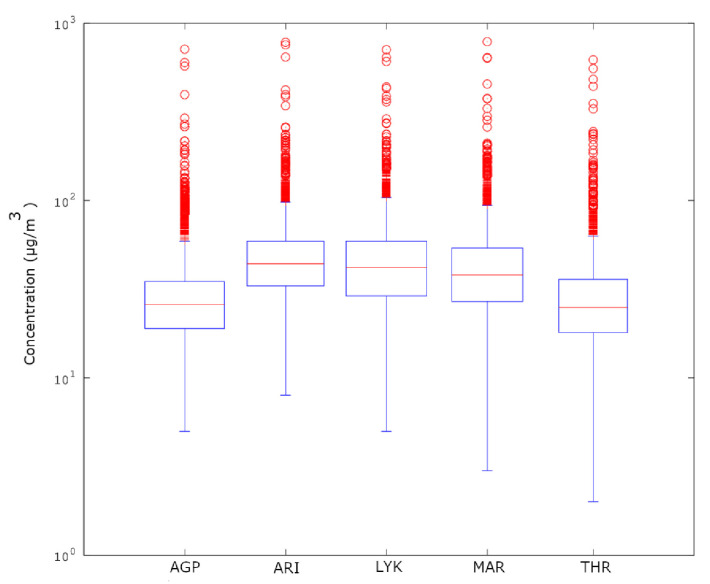
Box and whiskers plot of the full dataset PM_10_ concentrations in GAA in the period 2001–2010.

In the above consensus, it should be stressed that the decreasing tendency in the average PM_10_ values is also observed in Figure 5 and Figure 6 where the sliding window approach of step one is followed. The finer texture is due to the dense sliding window analysis. Despite that, the lumping process also delineates the stochastic trends. As already mentioned in Section 3.3, there are cases where the lumping process is advantageous. As can be observed from these figures, there is significant decreasing trend between Windows 1 and 4000 for the AGP (Figure 5a), THR (Figure 5c), ARI (Figure 6a) and MAR (Figure 6b) series and between Windows 1 and 3500 for the LYK (Figure 5b) series. However, when a whole year of data are included in the analysis, the partial trends are suppressed leading the several variance-inducing trends to diminish. All the above trends are presented integrally in Figure 7. This combination box and whiskers plot shows clearly that the PM_10_ data have many outliers and wide interquartile ranges (IQR). This is an alternative indication that the PM_10_ generation system is not only stochastic in nature. The same figure also leads to another observation noted in a very recent publication of the team [43]: ARI, LYK and MAR stations have similar behaviour. This behaviour can also be outlined by observing Figure 5b in comparison to Figure 6 and Figure 2b versus Figure 3. According to these observations, there is indication that the LYK should be characterised as UT and not as a SB.

**Figure 8:**
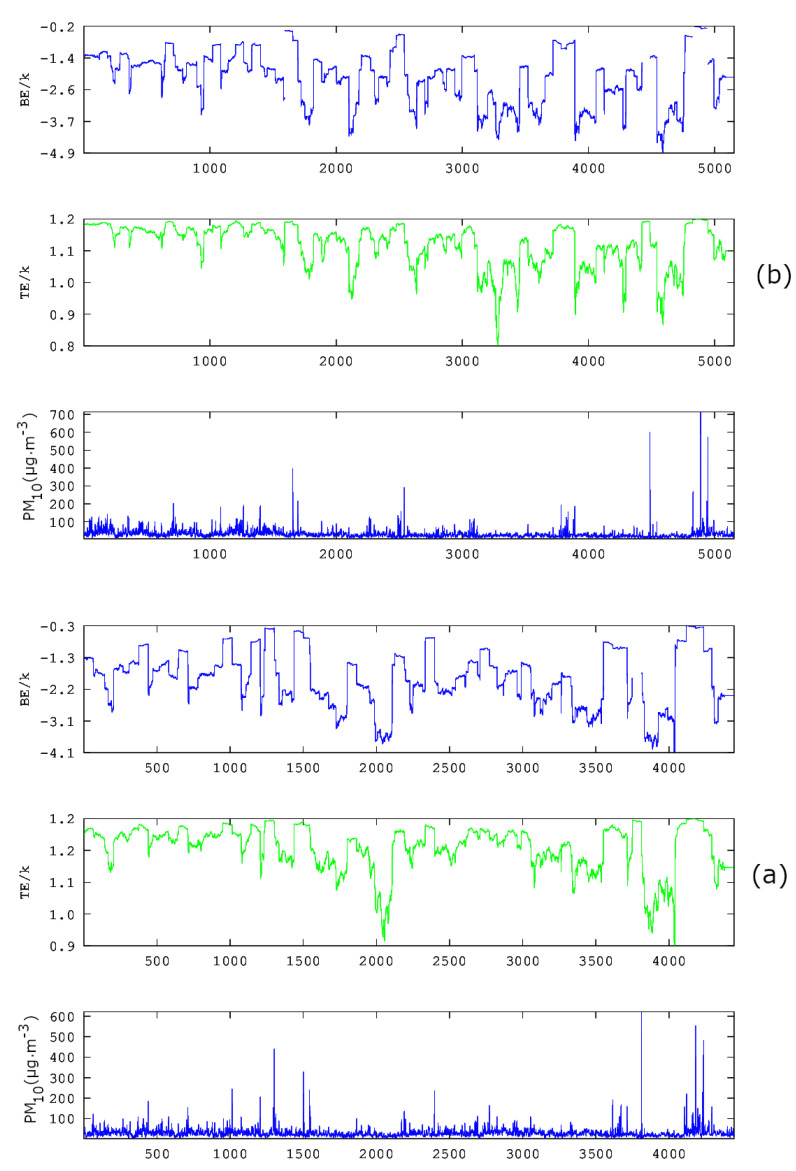
Time evolution of Boltzmann Entropy (BE) (light blue, top) and Tsallis Entropy (TE) (green, middle) in parallel to the original time series (dark blue, bottom): (**a**) AGP series; and (**b**) THR series. Analysis in segmented data of the time series for windows of length *n* = 64 with a step of 1. GAA, period 2001–2018. *k* is the Boltzmann’s constant. Average PM_10_ concentrations are in $\mu g/m^{3}$. The diagrams are produced with Octave Forge package.

**Figure 9:**
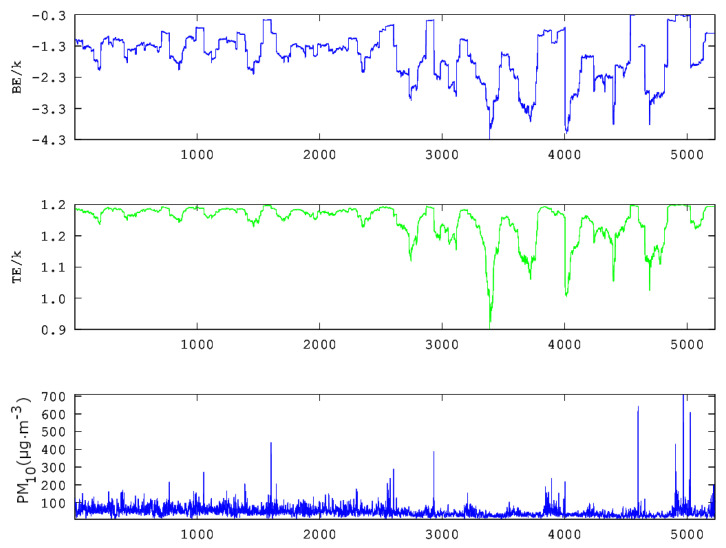
Time evolution of Boltzmann Entropy (BE) (light blue, top) and Tsallis Entropy (TE) (green, middle) in parallel to the original time series (dark blue, bottom) for the LYK series. Analysis in segmented data of the time series for windows of length *n* = 64 with a step of 1. GAA, period 2001–2018. *k* is the Boltzmann’s constant. Average PM_10_ concentrations are in $\mu g/m^{3}$. The diagrams are produced with Octave Forge package.

**Figure 10:**
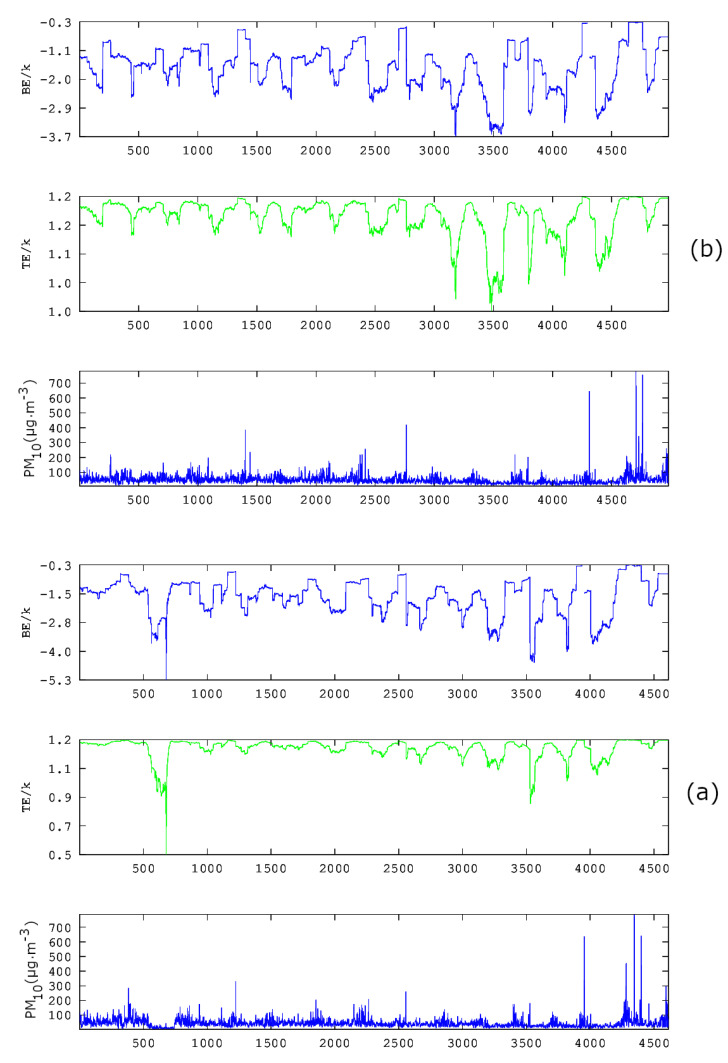
Time evolution of Boltzmann Entropy (BE) (light blue, top) and Tsallis Entropy (TE) (green, middle) in parallel to the original time series (dark blue, bottom): (**a**) ARI series; and (**b**) MAR series. Analysis in segmented data of the time series for windows of length *n* = 64 with a step of 1. GAA, period 2001–2018. *k* is the Boltzmann’s constant.Average PM_10_ concentrations are in $\mu g/m^{3}$. The diagrams are produced with Octave Forge package.

From the above data, it is evident that the statistical analysis explains partly the variations of the PM_10_ concentrations in GAA and, hence, may not provide a full outline of the involved physical mechanisms. This is because the related physical phenomena are not stochastic only. As analysed in recent publications of the team [36,37], chaos and long memory are present in various parts of the PM_10_ series. As described in Section 1 for urban pollution time series and in previous publications of the team for other environmental time series, the presence of chaos and long memory in time series may be associated with self-organised critical (SOC) behaviour and vice versa. In this consensus and following the discussion of Figure 2– Figure 6, Figure 8, Figure 9 and Figure 10 present the results of the entropy analysis versus time for windows of length *n* = 64 and a step of 1. The reader may recall that the use of the sliding window technique of these two figures is identical to the one of Figure 5 and Figure 6. As implemented in the analysis of the recent related publications of the team [36,37], the existing measurements are treated jointly, namely the missing values are neglected, because, as explained in these publications, this data treatment is advantageous for SOC analysis. For other types of analysis, e.g., for smart systems, other approximations are also valid [53].

The majority of the *n* = 64 sample windows of all examined time series exhibit medium to low variations of the Boltzmann entropy (BE) and Tsallis entropy (TE). Generally, this is an alternative indication that the PM_10_ system has stochastic behaviour, and, hence, it is in non-critical phases. For this reason, the statistical analysis of the data of these periods is adequate and provides useful outcomes. However, there are periods where BE, TE or both are considerably lower compared to the values of the remaining windows. Similar findings have been reported for the same time series in [36,37] through fractal and long-memory analysis of the data series. It is of importance that the periods of low BE and TE correspond to SOC phases of the system. These SOC phases have been associated with periods of high fractality and long memory [36,37] and are of significance for the environmental PM_10_ generating system. Indeed, BE is the fundamental measure of randomness [40]. Therefore, if BE is low, then the average amount of information necessary to predict a forthcoming window of length *n* = 64 is small. On the contrary, in the windows where the system is governed by stochastic processes, BE is elevated, and, hence, there is need for a large amount of information to predict future states. Moreover, there is a great uncertainty in predicting steps in the future provided the history of the present state of the system. There is another significant property of systems in SOC phases that differentiates their behaviour when compared to the stochastic ones. Importantly, the systems in SOC phases or, generally, in self-organisation define their future states not only from their present states but also by their long-term history [36,37]. These systems are non-Markovian in nature, contrary to the stochastic systems where their future is a probabilistic projection of their present states only. The above findings are also verified by the results of the TE. Tsallis entropy drops to lower values in certain windows. In these cases, there is a drop in the number of different kinds of patterns of the system [40]. This implies self-organisation. This finding in conjunction with the results of BE means that the states where BE or TE drop refer to SOC states, characteristic of a chaotic behaviour with high level of prediction [36,37]. It is important to also note that, especially Tsallis entropy, is very sensitive in recognising long-range interactions, long memory and multi-fractals [40]. The current outcomes, hence, imply the presence of fractality and long memory in the environmental PM_10_ generating system. This verifies the results of the previous studies of the team [36,37,40]. All the above show how important are the dynamics of entropy for the investigation of environmental time series by providing noteworthy measures of their complexity.

As already mentioned, the team has utilised, in four publications [36,37,38,39], combinations of 2–13 different chaotic techniques to analyse time series for hidden fractal and long-memory trends. Two of these publications referred to PM_10_ time series. As reported, it is not only the identification of critical fractal and long-memory epochs that provide valuable results, but rather the identification of common epochs by different techniques. The issue is also discussed in other publications of the team [50]. In the above consensus, a further step is implemented, namely the combination of the two entropic measures reported in Figure 8, Figure 9 and Figure 10. According to the data presented thus far, it is evident that the PM_10_ air-pollution system has both stochastic and long-lasting trends. Since the PM_10_ data exhibit both type of trends, it is proper that the two entropy analysis techniques are combined further with a stochastic type technique.

In the above sense, in [39], chaotic techniques are combined with the so-called relative concentration data, which refer to the residuals of the concentration data. According to the authors, the residual of each PM_10_ concentration is the term $residual\left(t\right)=c\left(t\right)-c_{ma}\left(t\right)$, where c(t) is the daily PM_10_ concentration and $c_{ma}\left(t\right)$ is the seven-day moving average. The daily concentrations (c(t)) of each station are indicated as PM_10_ in the first sub-plot of each one of three-column multi-plot of Figure 8, Figure 9 and Figure 10. As discussed by Alam et al. [39] (and the references therein), this technique has a long history in successfully delineating anomalous periods of stochastic data (e.g., radon in soil, groundwater and atmosphere). The anomalous periods are usually identified as those with residual(t) exceeding the limits of ±σ, ±2σ or even ±3σ, where σ is the standard deviation of the whole data. Following this viewpoint, the facts presented in the penultimate paragraph of the Introduction and the results presented thus far, it is evident that the PM_10_ air-pollution environmental system has both stochastic and long-lasting parts, the latter where entropy drops (see also discussion in previous publications of the team [40,41] and the references therein). In the consensus described above, the parameter residual(t) is, indeed, a measure of the stochastic tendencies of the PM_10_ time series. In this viewpoint, the critical limit for residual(t) within a certain window to characterise this as a stochastic anomaly could be selected as ±σ, ±2σ or even ±3σ. In the opinion of the authors, ±σ is adequate for the analysis. This is because the ±σ selection is a fair compromise between the several hidden trends of the data in comparison to the normal distribution trend, i.e. the trend needed so that σ is a meaningful measure of randomness-stochastic behaviour. Following this logic, if a daily PM_10_ relative concentration (residual(t)) exceeds ±σ, it is considered as an anomaly from a stochastic tendency (σ is the standard deviation of the whole data). Accepting the limit for the stochastic trends of the data, if cut-off limits for entropy (BE and TE) are set, as described in the previous publications of the team [36,37,38,39], the combination of the three techniques (one statistical and two entropic) can be implemented.

**Figure 11:**
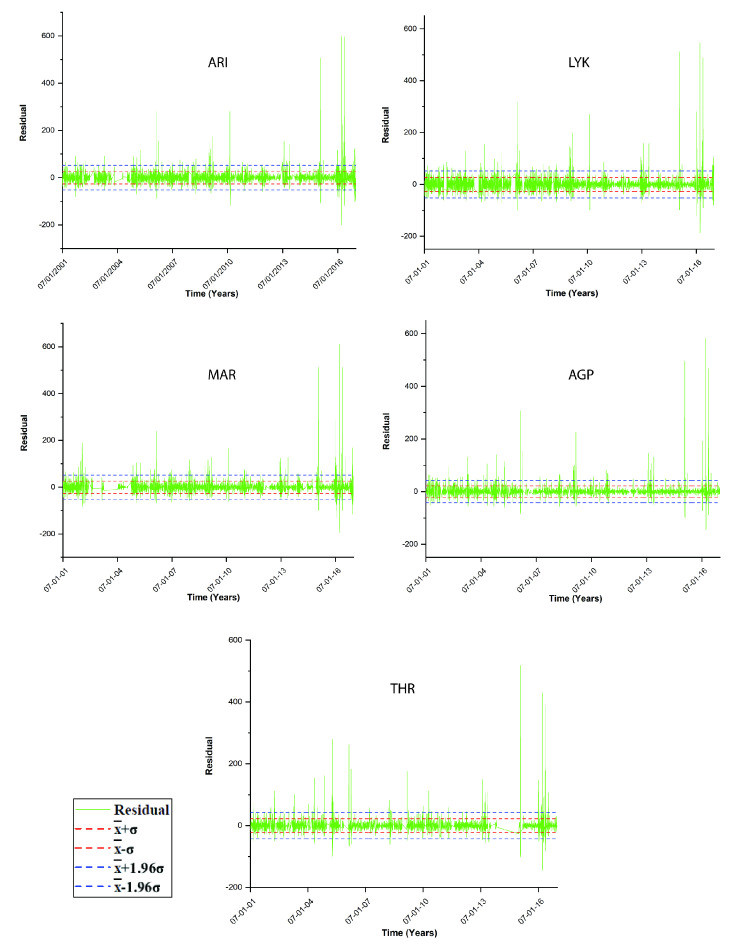
Time evolution of residual(t) of the PM_10_ concentration data as a function of time. GAA, period 2001–2018. *k* is the Boltzmann’s constant. Average PM_10_ concentrations are in $\mu g/m^{3}$. ±·σ and ±1.96·σ cut-off limits are presented as well.

Figure 11 presents the residual(t) as a function of time for all stations in Table 1. It is emphasised again that residual(t) is a linear function of the daily PM_10_ concentrations and that it is a measure of stochastic behaviour of the data. For integrity, both figures also present the ±1.96·σ, which is the value corresponding to the 95% confidence interval. The non-stochastic phases of the PM_10_ air-pollution system are those corresponding to out-of-thresholds values. Clearly, if the PM_10_ system might exhibit low-entropy trends, it has to be only in the non-stochastic parts, hence where it is out of the stochastic thresholds. Of course, this is only a necessary condition. The sufficient condition for a low entropy-high complexity state (also a low fractal state) is the one where the system is both out of stochastic behaviour and in a low entropy state. Towards this, the daily PM_10_ concentrations for which residual(t) is out of thresholds are extracted in ASCII format as a function of the time moments for which the exceedance is found. A similar procedure is followed for the exceedances of BE and TE of Figure 8, Figure 9 and Figure 10. The corresponding cut-off limits are selected as −2.0 for BE and 1.18 for TE. These limits are arbitrarily set as in the previous related publications [36,37,38,39] in accordance to the ASCII data of the temporal variations of BE and TE. This part of the analysis is described as Step 2 meta-analysis of the data. Thereafter, the out-of-thresholds time series are searched for common dates (Step 3 meta-analysis). Summarising the steps for the analysis are as follows: (**a**) Calculate TE, BE and residual time series. (b) Set cut-off limits and calculate the out-of-thresholds time series (Step 2 meta-analysis). (c) Locate the common dates for which BE, TE and residual out-of-thresholds time series are commonly addressed for every possible combination (Step 3 meta-analysis). For this study, the possible combinations are BE versus TE and RC, BE versus TE and TE versus RC.

Table 2 presents the common dates for every station of this study in accordance to the cut-off limits mentioned above and the meta-analysis of the ASCII data of the calculations of Figure 8, Figure 9, Figure 10 and Figure 11. As in the previous publications of the team, the presented dates are those that correspond to the start time of the window of analysis, considering that this describes the whole window. It is interesting that, under a similar calculation methodology (Step 2 and 3 meta-analyses) as in the previous related papers of the team [36,37], several common areas are identified. This is because, in this paper, only three techniques are joined together, whereas, in [37], 13 different techniques are joined. It should be emphasised that, as mentioned above, the identification of common dates via different techniques provides far more evidence regarding the justification that, during these dates, the PM_10_ environmental system is, indeed, in critical states (here SOC states). As analysed above and in the related papers of the reporting team, during these critical states, the PM_10_ system is in a chaotic and SOC regime that corresponds to an extreme solution path of the space phase volume, where the system behaves in a predictable non-Markovian manner, such that the present state of the system is not only determined by its previous states but also by its long history, and, most importantly, it determines also its future states, in a fractal and SOC behaviour, i.e., its presence interferes with its long-term history and future.

It can be observed that certain dates are commonly found by more than one station. For this reason, Table 3 presents only the dates that are commonly found by at least three stations. Six dates are commonly identified by four stations. For these dates, there is increased evidence that the underlying environmental system that generates the PM_10_ variations is in SOC states. This is a very significant finding because the dates of Table 3 show clearly that the SOC regime is common to at least three stations. That clearly indicates that are other mechanisms that lead the system to critical predictable states. As reported by Nikolopoulos et al. [37], such mechanisms are:

1. combined, continuously altering, power-law fractal or SOC state interactions of the PM_10_ air pollution system with several diverse sources of pollutants such as industrial processes, vehicular emissions and energy production from power stations, coupled with complicated physical and chemical processes [27,34];
2. different long-range temporal correlations for the small and large fluctuations of the PM_10_ time series [34,54], for example, different types of persistency or anti-persistency at the small and large time scales [28];
3. scale-free and self-affine fractal PM_10_ systems with the power-law scaling and long-term memory [54] recognised as a footprint of SOC behaviour [28];
4. non-linear dynamics governing the PM_10_ system defined by low-dimensional chaos [55]; and
5. exogenous forces (e.g., meteorological processes and photochemical reactions) can amplify or reduce the linear and non-linear mechanisms of the interactions among the ambient pollutants, which can further complicate the temporal structure of the dynamics of ambient pollutant concentrations, leading to complex behaviour of the air pollution system [56].

Most important, however, are the dates that are presented in Table 4. These are common dates during which the following constraints are simultaneously met for the PM_10_ system: (a) is out of stochastic behaviour; (b) shows low entropy values both for BE and TE; and (c) presents critical behaviour commonly found with different techniques (Points (a) and (b) from this study). This is an uncommon finding and should be emphasised. Of extreme importance are the first two dates. During these dates, 13 + 2 = 15 different techniques coincide (two from this study, BE and TE). For these two dates, there is very strong evidence that the PM_10_ system exhibits critical fractal and SOC behaviour. Date 3 of Table 4 is of lesser significance compared to the other two, because there is coincidence only from four techniques (including the two of this study, BE and TE). The reader should emphasise the fact that it is not trivial to find SOC and fractal traces simultaneously. In the related literature, the analysis is usually confined to visual observations and some statistical analysis of the data. Even in the literature that focuses on fractal and SOC trends of environmental data (please see the Introduction and the references cited therein), to the knowledge of the authors, the majority of the related papers report findings with some techniques, but not in combination. The fact that the findings of this study, have coincidences with the outcomes of previous papers of the team justifies the validity of the whole approach, and, most significantly, it is in agreement to the related theory that predicts coincidence between critical fractal and SOC trends. It should be noted that this interesting finding is only addressed in the three dates of Table 4 and not in the others of Table 3. This can be attributed to the multi-parameter nature of the related processes (combined effect of, traffic, industry, wind, temperature gradients, rainfall alterations, particulate dust particles, heating of houses, etc.). The non-linear inter-relations of these related parameters lead the PM_10_ system out of stochastic behaviour force it to enter into non-linearity and, in some cases, deterministic chaos and SOC states. The reader should also recall that the dates of Table 4 correspond to the beginning dates of segmented-windowed data, therefore, the coincidence is wider in time.

It is evident from the above that the first two dates of Table 4 are the most significant. For this reason, block entropy analysis is further applied in the two-month windows that correspond to these dates. As implied in Section 3.5, it is significant to emphasise in relation that block entropy provides the local SOC trends of the time series. This is because the actual local variations are re-organised as ones and zeros and, thereafter, as words of different letters. As discussed in the references cited in Section 3.5, the technique is very advantageous when comparing time series segments of different hidden-internal patterns. To allow comparisons of the aforementioned two-month segment areas with other parts of the PM_10_ data, it is crucial to locate proper PM_10_ parts. The window parts that correspond to the dates of Table 3, but excluding the ones of Table 4, can be located for which the PM_10_ system: (**a**) is out of stochastic behaviour; and (b) exhibits some kind of SOC trends. To generate a wide window area, all the windows of Table 3, except those of Table 4, are joined together in an area, hereafter called the "net" area. To discriminate this with the previous areas, the windows-areas of the first two dates of Table 4 are hereafter called "Area 1" and "Area 2". Table 5 presents the results of block entropy analysis utilising the same metrics as in the entropy analysis versus time, i.e., Boltzmann block entropy (BE) and Tsallis block entropy (TE).

All values of Table 5 have a ±0.007 calculation uncertainty. This was estimated by biasing the start of the window of calculation by ±10 samples, where the value of 10 was arbitrarily selected. Observing Table 5, the following information can be supported: (a) BE and TE block entropy values saturate above *n* = 5 letters. This saturation has been reported by others [51]. (b) The five different stations of the investigation exhibit differentiations in the BE and TE block entropy values; however, the different values are of similar ranges. (c) TE has significantly lower values compared to BE. The most significant observation is the following: (d) BE and TE entropy values of the net-area are significantly higher than the ones of Areas 1 and 2. This implies a fact of extreme importance. The self-organisation of the PM_10_ system in Areas 1 and 2 is significantly higher than the one of the net area. The reader should recall that the net area also has SOC patterns. Therefore, this identification of higher organisation in Areas 1 and 2 provides very well based evidence that the system in Areas 1 and 2 is, actually, in the most significant SOC states. Since these areas are also identified in the previous publications of the team, there are 15 different methodologies that provide this evidence. It is very important that in Areas 1 and 2 the system is not only in chaos and SOC states but in highly predictable states. Therefore, it is in solutions where its presence is defined not only by its past but also by its long-term history, and, significantly for non-Markovian systems, it defines its long-term future. This important finding should be emphasised and is a significant outcome of this research.

Despite the increased evidence found for the two dates of Areas 1 and 2, all the dates of Table 3 are important, as well as the results of other dates reported in the previous publications of the team [36,37]. The above facts are indicative of the advancement of the new methodology described in the previous publications [36,37,38,39]. It is significant that the technique has been checked in other environmental systems (electromagnetic radiation disturbances and radon in soil variations). Three related publications of the team clearly show that the PM_10_ environmental system can be described by similar techniques. It is also important to emphasise that this is not the first research to address fractal and SOC trends in PM_10_ air-pollution variations. As presented in the several papers cited in the Section 1, many researchers have reported such trends. However, this is the first time to combine so many different techniques and locate the most important time series parts found from very lengthy time series. Of course the fact that only two areas are commonly found does not lower the validity of the reported data, because there are many more areas with SOC trends given in Table 3. Moreover, the paper analyses the stochastic trends of the data in a systematic manner. Importantly, many areas of the whole time series exhibit stochastic behaviour, and this is the trivial hypothesis when working with environmental air-pollution time series. However, it is also studied very systematically that there are also non-stochastic fractal and SOC phases which cannot be neglected and should be taken into account in the related studies. Finalising, it is an issue of further research to investigate more data and other air-pollution time series.

## 5. Conclusions

In the following, the most significant findings of the present paper are summarised:

1. Statistical and entropy analysis methods are employed for the study of a 17-year PM_10_ time series recorded from five stations in Athens, Greece. The purpose is to investigate further the stochastic trends of the series and explore if non-stochastic periods of low entropy values exist.
2. The stochastic trends are analysed in monthly, two-month and annual windows via lumping and sliding windows. Decreasing time-evolution trends were found: (a) between Windows 40 and 130 for n=32 and a step of 32 (lumping process); and (b) between Windows 1 and 4000 for the AGP, THR, ARI and MAR series and between Windows 1 and 3500 for the LYK series, all for n=64 and a step of 1 (sliding window process). For Case (b), periods with high variance, skewness above 1 and kurtosis well above 3 are found. Deviations from the Gaussian distribution are addressed in various parts and, therefore, non-statistical behaviour. However, when the data are analysed for n=365 and a step of 365 (annual analysis through lumping), they follow the normal distribution. Several outliers are also found. ARI, LYK and MAR stations have similar IQR behaviour, indicating that LYK should be characterised as UT. The discrepancies from the stochastic behaviour are attributed to the multiple facets involved in physical procedures. The related mechanisms are discussed.
3. Self organisation is investigated via Boltzmann and Tsallis entropy. Sliding windows of n=64 and a step of 1 are employed and symbolic dynamics in selected parts. The majority of the n=64 windows of all examined time series exhibit medium to low variations of both entropy values. Several low entropy parts are identified with Boltzmann entropy over Boltzmann constant below -2.0 entropy and Tsallis entropy over the Boltzmann constant below 1.18. The implications of the findings are discussed.
4. A previously published combination two-step method is utilised to locate areas for which the PM_10_ system is out of stochastic behaviour and, simultaneously, exhibits critical self-organised patterns.According to previous publications, the non-stochastic periods are taken those with residual(t) out of ±σ. These periods are located and extracted to ASCII data. The two-step methodology is applied to those periods and the critical entropy periods of Conclusion (b) above. Sixty-six different periods of two-month duration are found in the time series for various dates. From these, nine periods are common to at least three different stations. This is very significant because it is associated with internal dynamics of the GAA basin.
5. Searching for enhanced evidence of SOC and fractal trends, as well as long memory, the findings of this study are further compared to previous publications for the same series. Two areas are found for which the series is non-stochastic and exhibit, simultaneously, fractal, long-memory and self-organisation patterns through a combination of 15 different fractal and SOC analysis techniques.
6. In the two most significant areas, block entropy analysis is further applied. For comparison, block entropy is also utilised in the remaining identified parts with SOC patterns. Block entropy values are in the range 0.650–2.924 for words of 2–7 letters. Block entropy saturates above five letters. Significantly lower block entropy values are found for the two areas identified with the combination of 15 techniques, compared to the remaining areas.
7. This is the first time to utilise entropy analysis for PM_10_ series and, importantly, in combination with results from previously published fractal methods. This combination approach is expected to be applied in the future to other environmental series, e.g., in environmental ozone and PM_2.5_ series, as well as in more seismic related series, such as radon in groundwater and soil and MHz−kHz environmental electromagnetic disturbances. Despite the limited number of critical windows found, the enhanced evidence, on the one hand, and the increased outcomes, on the other hand, which are expected to be received in more dense time series, make the present approach a significant study tool. Future expectations are the extent of the methodology with multifractal techniques to account for the multifractality found in nature, as well as combining the present techniques with more advanced statistical approaches.

## Figures and Tables

**Table 1:** The characteristics of the examined air pollution monitoring stations within the GAA. Abbr., abbreviation; Alt., altitude above sea level (a.s.I); D.C., data completeness.

Monitoring Station	Abbr.	Longitude	Latitude	Alt. (m)	Characterisation	D.C.
Aristotelous	ARI	23°43’39"	37°59’16"	75	Urban-Traffic	85.8%
Lykovrissi	LYK	23°47’19"	38°04’04"	234	Suburban-Background	89.2%
Maroussi	MAR	23°47’14”	38°01’51”	170	Urban-Traffic	82.5%
Agia Paraskevi	AGP	23°49’09”	37°59’42”	290	Suburban-Background	88.7%
Thrakomakedones	THR	23°45’29’	38°08’36”	550	Suburban-Background	77.2%

**Table 2:** Results of the common areas of all investigated stations exhibiting simultaneously: (**a**) residual(t) out of ±σ; (**b**) BE value below 2.0; and (**c**) TE below 1.18. i/i represents the index value of the common date of each station.

Monitoring Station	i/i	Date
AGP	1	2005/12/3
	2	2005/12/5
	3	2007/3/24
	4	2007/3/25
	5	2007/7/28
	5	2007/11/6
	6	2009/4/4
	7	2009/4/6
	7	2010/6/7
	8	2010/12/28
	9	2011/6/9
	10	2013/5/31
	11	2013/6/1
	12	2013/6/2
	13	2013/6/3
	14	2013/6/4
	15	2014/6/26
	16	2014/6/27
	17	2014/10/19
	17	2015/1/8
	18	2015/2/6
	19	2015/2/2
	20	2016/5/13
	20	2016/7/17
ARI	1	2007/3/25
	2	2009/4/4
	3	2009/4/6
	4	2010/3/5
	5	2010/3/6
	6	2010/3/7
	7	2010/3/8
	8	2014/6/26
	9	2014/6/27
	10	2015/2/6
	11	2015/2/2
	11	2016/5/13
	12	2016/7/17
LYK	5	2007/7/28
	1	2009/4/4
	2	2010/2/22
	3	2010/2/23
	4	2010/2/25
	5	2010/6/7
	6	2014/6/26
	7	2014/6/27
	8	2015/1/8
	9	2016/5/23
	10	2016/5/24
	11	2016/5/25
	12	2016/5/27
MAR	1	2007/3/24
	2	2007/3/25
	3	2007/6/14
	5	2007/7/28
	3	2009/4/4
	4	2009/4/6
	5	2010/2/22
	6	2010/2/23
	7	2010/6/7
	8	2014/6/26
	9	2014/6/27
	10	2015/1/8
	11	2015/1/22
	12	2015/2/6
	13	2015/2/2
	14	2015/2/7
	15	2016/7/22
THR	1	2009/4/4
	2	2009/4/6
	3	2010/12/28
	4	2011/6/9
	5	2015/2/6
	6	2015/2/2
	7	2016/7/17

**Table 3:** Results of the areas of Table 2 that are commonly found by at least three stations. All investigated dates exhibit simultaneously: (a) residual(t) out of ±σ; (b) BE value below 2.0; and (c) TE below 1.18. i/i represents the index value of the common dates.

i/i	Date	Monitoring Stations
1.	2007/3/25	AGP, ARI, MAR
	2007/7/28	AGP, LYK, MAR
2.	2009/4/4	AGP, ARI, LYK, THR
3.	2009/4/6	AGP, ARI, MAR, THR
4.	2010/6/7	AGP, LYK, MAR
5.	2014/6/26	AGP, ARI, LYK, MAR
6.	2014/6/27	AGP, ARI, LYK, MAR
	2015/1/8	AGP, LYK, MAR
7.	2015/2/6	AGP, ARI, MAR, THR
8.	2015/2/2	AGP, MAR, THR
9.	2016/7/7	AGP, ARI, THR

**Table 4:** Results of the areas of Table 3 that are commonly addressed with the previous related papers of the team with other techniques.

i/i	Date	Monitoring Stations	Techniques	Publication
1.	2007/7/28	AGP, LYK, MAR	13 different fractal techniques	Nikolopoulos et al. [36]
2.	2010/6/7	AGP, LYK, MAR	13 different fractal techniques	Nikolopoulos et al. [36]
3.	2015/1/8	AGP, LYK, MAR	DFA & RS analysis	Nikolopoulos et al. [37]

**Table 5:** Results of the block entropy analysis of selected areas of the PM_10_ time series. Clarifications are written in text.

		Area 1		Area 2		Area Net	
Station	Letters	BE	TE	BE	TE	BE	TE
AGP	2	1.095	0.682	1.108	0.650	1.231	0.743
	3	1.679	0.896	1.143	0.699	1.847	0.921
	4	1.859	0.927	1.398	0.701	2.364	1.005
	5	2.095	0.997	1.834	0.939	2.847	1.064
	6	2.095	0.997	1.835	0.938	2.843	1.069
	7	2.097	0.996	1.832	0.943	2.846	1.062
ARI	2	1.196	0.731	1.206	0.735	1.328	0.792
	3	1.665	0.891	1.413	0.801	1.828	0.910
	4	2.119	1.009	1.678	0.900	2.442	1.039
	5	2.109	0.997	1.945	0.986	2.895	1.090
	6	2.111	0.998	1.946	0.987	2.897	1.091
	7	2.112	0.998	1.947	0.987	2.898	1.091
LYK	2	1.045	0.651	1.305	0.789	1.267	0.757
	3	1.708	0.892	1.751	0.907	1.887	0.936
	4	1.864	0.957	2.026	0.975	2.396	1.017
	5	1.981	0.976	2.138	1.015	2.924	1.094
	6	1.982	0.977	2.139	1.015	2.295	1.096
	7	1.981	0.978	2.141	1.016	2.297	1.097
MAR	2	1.227	0.746	1.323	0.802	1.231	0.740
	3	1.821	0.943	1.775	0.924	1.781	0.892
	4	2.211	1.024	2.211	1.024	2.281	0.986
	5	2.369	1.057	2.222	0.993	2.534	1.035
	6	2.371	1.059	2.221	0.995	2.532	1.037
	7	2.369	1.062	2.223	0.997	2.536	1.037
THR	2	1.208	0.703	1.157	0.695	1.243	0.754
	3	1.878	0.941	1.478	0.809	1.829	0.919
	4	1.841	0.946	1.925	0.974	2.397	1.031
	5	1.981	0.976	2.025	0.993	2.849	1.068
	6	1.983	0.977	2.027	0.991	2.856	1.069
	7	1.984	0.975	2.029	0.994	2.842	1.067
